# An Approximate GEMM Unit for Energy-Efficient Object Detection

**DOI:** 10.3390/s21124195

**Published:** 2021-06-18

**Authors:** Ratko Pilipović, Vladimir Risojević, Janko Božič, Patricio Bulić, Uroš Lotrič

**Affiliations:** 1 Faculty of Computer and Information Science, University of Ljubljana, 1000 Ljubljana, Slovenia; patricio.bulic@fri.uni-lj.si (P.B.); uros.lotric@fri.uni-lj.si (U.L.); 2 Faculty of Electrical Engineering, University of Banja Luka, 78000 Banja Luka, Bosnia and Herzegovina; vladimir.risojevic@etf.unibl.org; 3 Biotechnical Faculty, University of Ljubljana, 1000 Ljubljana, Slovenia; janko.bozic@bf.uni-lj.si

**Keywords:** approximate general matrix multiplication, GEMM, tensor core, matrix core, approximate computing, approximate multipliers, convolutional neural networks, energy-efficient processing, object detection, YOLOv4-tiny, honeybee detection

## Abstract

Edge computing brings artificial intelligence algorithms and graphics processing units closer to data sources, making autonomy and energy-efficient processing vital for their design. Approximate computing has emerged as a popular strategy for energy-efficient circuit design, where the challenge is to achieve the best tradeoff between design efficiency and accuracy. The essential operation in artificial intelligence algorithms is the general matrix multiplication (GEMM) operation comprised of matrix multiplication and accumulation. This paper presents an approximate general matrix multiplication (AGEMM) unit that employs approximate multipliers to perform matrix–matrix operations on four-by-four matrices given in sixteen-bit signed fixed-point format. The synthesis of the proposed AGEMM unit to the 45 nm Nangate Open Cell Library revealed that it consumed only up to 36% of the area and 25% of the energy required by the exact general matrix multiplication unit. The AGEMM unit is ideally suited to convolutional neural networks, which can adapt to the error induced in the computation. We evaluated the AGEMM units’ usability for honeybee detection with the YOLOv4-tiny convolutional neural network. The results implied that we can deploy the AGEMM units in convolutional neural networks without noticeable performance degradation. Moreover, the AGEMM unit’s employment can lead to more area- and energy-efficient convolutional neural network processing, which in turn could prolong sensors’ and edge nodes’ autonomy.

## 1. Introduction

Artificial-intelligence-powered edge computing has brought complex processing devices closer to the data source, compromising their autonomy [1]. As the data processing on edge devices becomes computationally complex and power demanding, we have to pursue energy-efficient processing.

Object detection is a challenging computer vision task that is comprised of the localization and classification of objects [2,3] and thus helps to provide a proper understanding of an image. Traditional object detection models include informative region selection, the extraction of features, and classification. However, during the last decade, deep-neural-network-based detection models that merge the above steps and are trained on large databases of labeled images have evolved as state-of-the-art approaches for object detection [4,5,6].

Despite recent advances, the detection of small, fast-moving objects, such as honeybees, where processing speed plays a critical role, remains a challenging task. In [7,8,9,10,11], several systems for identifying honeybees and pollen loads, as well as monitoring the health conditions in a beehive were proposed. Babic et al. [7] proposed a Raspberry Pi-based system for the detection of pollen-bearing bees. The authors in [8,10] proposed a method for automatic monitoring of honeybees’ activity outside of the hive using a video captured by unmanned aerial vehicles. The study [9] presented a portable computer vision system that could monitor the infestation level of the Varroa destructor mite in a beehive by recording a video sequence of honeybees. Finally, the most recent study [11] was the first to use deep neural network object detectors implemented on graphics processing units for Varroa destructor mite detection on a honeybee. However, all these solutions were based on offline processing of the recorded images or videos and lacked permanent monitoring performed near beehives, commonly without a power supply, ensured only by a long-term autonomy device.

Accelerating deep neural network processing in edge computing using energy-efficient platforms is an important goal [12,13,14,15,16,17,18,19,20,21,22,23,24,25,26,27]. Currently, most object detection and classification models are carried out in graphics processing units. In edge computing, a platform containing a rather powerful graphics processing unit cannot meet the requirements of being small, operating in real time, and consuming little power. Therefore, many lightweight approaches with low-power consumption and low-computational performance have emerged recently. A few dedicated neural network accelerators have been implemented on FPGA hardware platforms [12,14,17,21,23], while several authors proposed ASIC-based neural network accelerators [13,15,16,18,19,22]. Samimi et al. [20] proposed a technique based on the residue number system to improve the energy efficiency of deep neural network processing. Si et al. proposed computing-in-memory as a promising approach to reduce the latency and improve the energy efficiency of deep neural network edge processors [24]. Scalable convolutional blocks were proposed in [25] to easily balance processing speed and accuracy in consideration of the computing power of various edge computing devices. The scalable and fast lightweight YOLO detector was designed using these scalable convolutional blocks and tested on various graphics processing units. The paper [26] provided an overview of the recent hardware and algorithm co-design schemes enabling efficient processing of deep neural networks. The authors in [27] proposed a novel real-time architecture and data flow by decomposing multiplications down to the bit level and pruning identical computations without reducing the accuracy of deep neural networks.

Deep neural network models are strong at generalizing the knowledge gained during the training. Adequately trained models are more error resilient with a lessened need for the accuracy of the results and computation, making them perfect candidates for approximate computing. Approximate computing is a new paradigm where an acceptable error is induced in the computing to achieve more energy-efficient processing [28,29,30,31,32,33]. It has been introduced at different system levels [34,35,36,37,38,39,40,41,42,43,44,45], and a large number of approximate arithmetic circuits have been designed to save chip area and energy [35,38,46,47,48,49,50,51]. Multiplication is a very common, but expensive operation, with exact multipliers being large circuits that consume a significant amount of energy. Various approximate multipliers have been proposed in recent years [52,53,54,55,56,57,58,59,60,61,62]. Many studies reported that approximate multipliers behave well in neural network processing [56,59,60,61,63,64,65].

Deep neural network models with many convolutional and fully connected layers must perform numerous matrix–matrix operations involving a vast number of arithmetic operations and external memory accesses. We can efficiently describe the most demanding matrix multiplications with the general matrix multiplication (GEMM) operation comprised of matrix multiplication and accumulation. Hence, instead of designing dedicated neural network accelerators, the trend is to introduce the GEMM operation accelerating hardware units into graphics processing units. For example, Nvidia introduced a dedicated hardware unit called the tensor core in 2017 with the Volta architecture [66]. Yan et al. [67] described how tensor cores work in great detail, including the instructions used and the registers and data layout required. Each tensor core consumes two four-by-four matrices with half-precision floating-point operands and computes their multiplication result in one clock cycle.

Edge computing devices mainly uses pretrained deep neural networks for inference. For efficient processing in edge devices, Nvidia has prepared the Jetson Xavier NX module with only 48 tensor cores, as opposed to the mainstream Tesla V100 graphics processing unit with 640 tensor cores [68]. Besides being less demanding, deep neural network computing in the inference step can also be less accurate than in the learning step, anticipating that the computing units in edge devices can be further simplified.

In pursuit of the long autonomy of the edge devices that perform demanding real-time processing, we proposed an approximate general matrix multiplication (AGEMM) unit. It combines the GEMM operation and approximate multipliers into a design that delivers high throughput and energy efficiency. Contrary to the approaches above, which mainly followed the exact computation, the AGEMM unit intentionally introduces some error in the computation to further accelerate the performance of edge devices. Additionally, we strove not to tailor the design to a specific deep neural network model, but for more general usage in deep neural networks or other applications. The application of the AGEMM unit in convolutional neural network processing should significantly provide benefits in terms of speed, energy, and area consumption at the expense of reduced accuracy in matrix multiplication. We anticipate that the reduction of the accuracy will not have significant detrimental effects on the performance of an object detector based on deep neural networks.

In the rest of the paper, we first provide some background on convolutional neural network processing and provide an overview the most recent approximate multipliers. Section 3 presents the hardware design of the AGEMM unit and the synthesis result, including the propagation delay, area, and energy consumption. In Section 4, we consider a honeybee detection application by assessing the applicability of the AGEMM unit in terms of speed and object detection accuracy. Finally, we conclude the paper with the main findings.

## 2. Background

Our goal was to design an energy-efficient GEMM unit that would enable object detection in real time. The design heavily relied on the chosen object detection model and the utilized approximate arithmetic circuits. This section provides the reasoning behind selecting the YOLOv4-tiny convolutional neural network model and a set of the approximate multipliers used as building blocks in the design of our unit.

### 2.1. Convolutional Neural Network YOLOv4-Tiny

State-of-the-art object detectors are mainly of two types: two-stage detectors and single-stage detectors [2]. The two-stage detectors generate regions of interests in the first stage and perform bounding box regression and object classification in the second stage. Some detectors belonging to this group are the region convolutional neural network (R-CNN) [69], Faster R-CNN [70], and the feature pyramid network (FPN) [71]. The two-stage detectors include various correlated phases such as region proposal generation, feature extraction using convolutional neural networks, bounding box regression, and classification, which are trained separately. The single-stage detectors address the complexity of the two-stage detectors and combine all phases into an end-to-end model predicting the bounding boxes and class probabilities from an image in one pass. Two-stage detectors possess high accuracy rates, but are slow, while single-stage detectors achieve lower accuracy, but work faster [72]. Widely used single-stage detectors are the you look only once detector (YOLO) [73], the Single-shot multi-box detector (SSD) [74], and RetinaNET [75]. Even though the initial YOLOv1 detector is inferior to the SSD and RetinaNET detectors, the YOLO family of detectors has been continuously evolving and upgraded with the models improved in terms of accuracy and speed, resulting in the best balance between accuracy and execution time among the mentioned single-stage detectors [2].

The YOLO family of detectors divides an image into multiscale regions and outputs bounding box and class probabilities for each region. One of the latest models is YOLOv4 [76] with a notable increase in object detection performance and less computational expense compared to other similar solutions. Despite the improvements, the YOLOv4 model is still computationally too demanding for real-time processing in edge devices. The YOLOv4-tiny model [77] is a lightweight version of the YOLOv4 model, which uses a compressed backbone for the two-scale feature extraction and an object-detection head with an anchor-based object bounding box predictor followed by multiscale object classification. In the description that follows, we focus on the computationally intensive backbone only.

**Figure 1:**
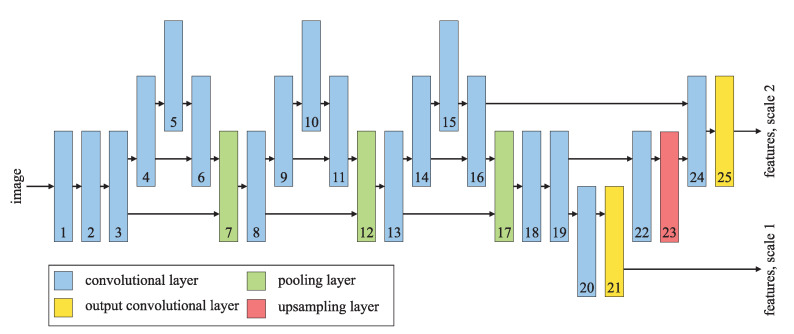
The backbone of the YOLOv4-tiny convolutional neural network [77].

The backbone illustrated in Figure 1 consists of a series of convolutional layers, combined with some pooling layers and an upsampling layer. Each layer operates on a series of two-dimensional feature maps or channels, which form a three-dimensional tensor. An input tensor is convolved with several multichannel filters and passed through the activation function to obtain an output tensor. The vast number of multiplications needed to compute the convolutions makes the backbone computationally demanding. The pooling layers reduce the size of each channel by taking the maximum of a set of neighboring elements, while the upsampling layer enlarges each channel by repeating the existing elements. Neither of them involves multiplication.

### 2.2. Approximate Multipliers

The approximate multiplier design focuses mainly on fixed-point operands, as floating-point operands with exponent handling add additional complexity to the multiplier circuitry. Although the range of values we can represent in the fixed-point format is smaller than in the floating-point format with the same operand width, it is sufficient for many applications. Wider operands could bring additional accuracy, but also an additional burden when transferring them from memory. For example, Nvidia uses 16-bit half-precision floating-point operands in its Volta tensor cores [66]. The majority of the state-of-the-art approximate multiplier designs focus on 16-bit operands. Hence, we opted for sixteen-bit designs, which are more accurate and address a broader range of applications than eight-bit approximate multipliers.

In the approximate multiplier design, we balanced accuracy and energy efficiency to suit the application’s needs. Various approximate multipliers have been proposed in recent years [54,55,56,57,58,59,60,61,62]. Approximate multipliers follow one of three design strategies: the approximate logarithmic design, the approximate nonlogarithmic design, and the hybrid design. Approximate logarithmic multipliers deliver a more straightforward design, but exhibit significantly higher computational error, while approximate nonlogarithmic multipliers have a lower computational error with the price of higher design complexity [54,61]. The hybrid multipliers combine both design strategies to balance accuracy and design complexity.

Approximate logarithmic multipliers rely on the addition of the approximate operands’ logarithms. Liu et al. [54] proposed the unsigned logarithmic ALM-SOA multiplier, which uses a truncated binary logarithm converter and a set-one-adder for the addition of logarithms to compensate the negative errors. Kim et al. [56] proposed the signed logarithmic multiplier Mitchell-trunc8-C1, which keeps only eight upper bits of the mantissa in the logarithmic representation of the input operands. The unsigned logarithmic ILM-AA multiplier with an approximate adder, proposed by Ansari et al. [59], further improves the error characteristics of the previously proposed designs. The dynamic range approximate logarithmic multiplier DR-ALM5 proposed by Yin et al. [57] dynamically truncates the input operands and thus uses smaller bit-width logarithmic and antilogarithmic converters and adders to generate the product. Pilipović et al. [61] proposed an approximate logarithmic multiplier with two-stage operand trimming (TL16-8/4), which trims the least significant parts of the input operands in the first stage and the mantissas of the obtained operands’ approximations in the second stage.

Approximate nonlogarithmic multipliers use the Booth algorithm to simplify the partial product generation stage and addition. In the RAD1024 multiplier [55], the input operand is divided into the upper part encoded using the radix-4 encoding, and the lower part of $\log_{2}1024$ bits, approximately encoded with the radix-1024 encoding. The HLR-BM2 multiplier [58] uses radix-8 encoding and approximates the ±3 multiplicands to their nearest power of two, such that the errors complement each other.

The hybrid multipliers LOBO12-12/8 [60] and HRALM3 [62] combine radix-4 Booth encoding and logarithmic product approximation to achieve a good tradeoff between accuracy and design efficiency.

**Figure 2:**
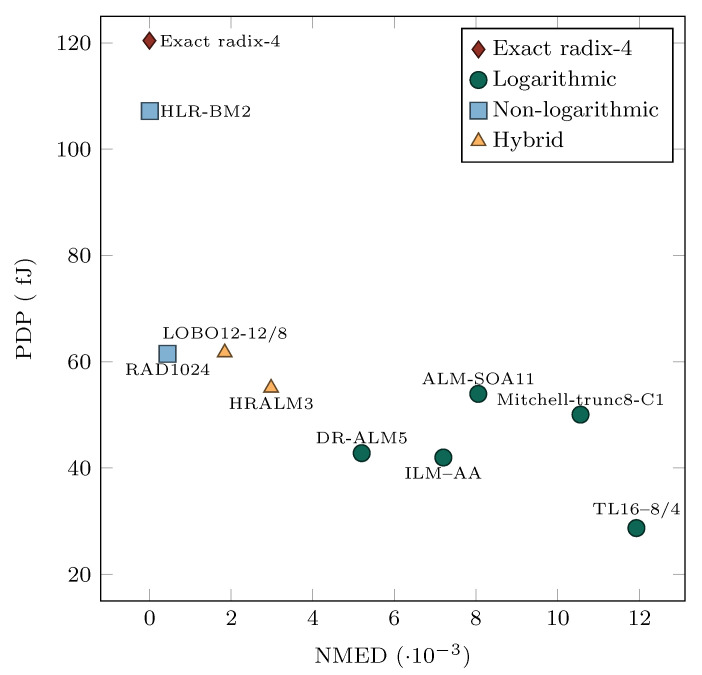
Comparison of approximate multipliers in terms of the power delay product (PDP) and normalized mean error distance (NMED).

The scatter plot in Figure 2, generated from the data obtained in [62], summarizes some of the state-of-the-art approximate multipliers in terms of standard comparison measures: power delay product (PDP) and normalized mean error distance (NMED) [61]. We can observe that more accurate nonlogarithmic multipliers consume more energy than logarithmic multipliers. However, the latter bring a more energy-efficient design at the price of lower accuracy. The hybrid multipliers sit in between both groups.

This paper used the existing state-of-the-art approximate multipliers DR-ALM5, TL16-8/4, RAD1024, and HRALM3 to implement and evaluate the approximate GEMM unit. With this selection of multipliers, we aimed to cover all design strategies and a broad spectrum of accuracy and energy efficiency.

## 3. An Approximate General Matrix Multiply Unit

**Figure 3:**
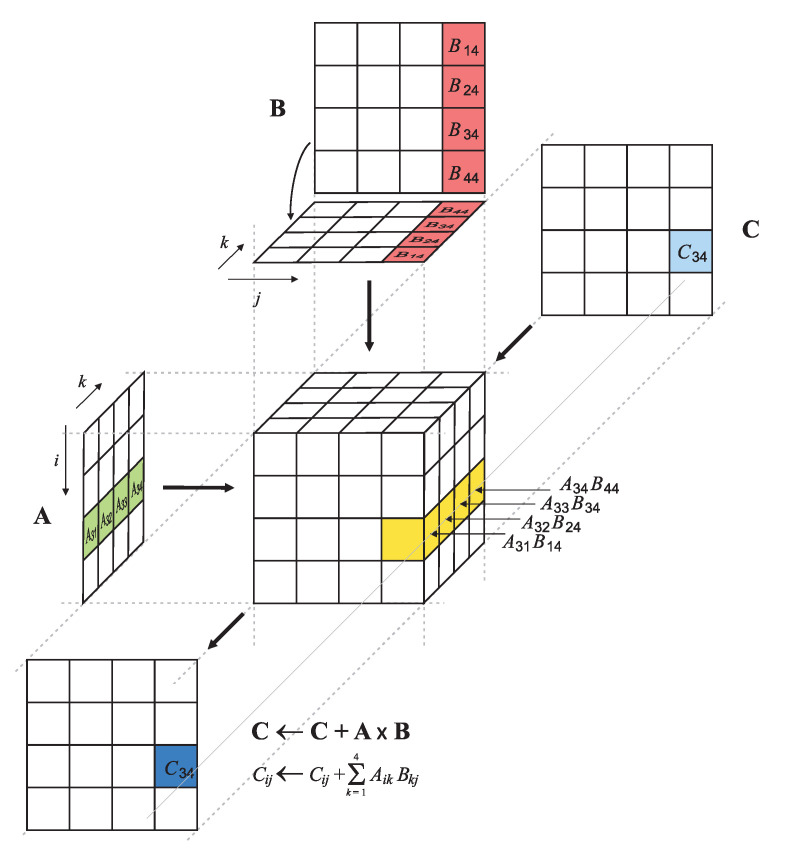
The general matrix multiply (GEMM) operation.

General matrix multiply (GEMM) is a standard operation in linear algebra, machine learning, statistics, and many other domains and serves as a core building block for deep learning computations [78,79,80]. The GEMM operation [67], illustrated in Figure 3,

(1) C←C+A×B,

adds the product of the *p*-by-*q* matrix A and the *q*-by-*r* matrix B to the *p*-by-*r* matrix C involving pqr multiplications and pqr additions.

### 3.1. The Hardware Implementation

A dedicated GEMM hardware unit executes the GEMM operation. Although the sizes of the matrices were arbitrary, we strove to use the GEMM unit efficiently and therefore kept the values low by setting p=q=r=4.

The basic building block of the GEMM unit is a multiply-accumulate (MAC) unit, which updates the scalar operand *c* with the product of the scalar operands *a* and *b*,

(2) c←c+ab.

**Figure 4:**
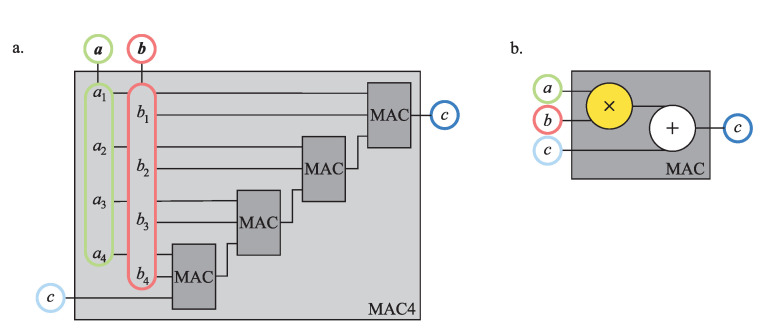
The MAC4 unit (**a**) composed of four MAC units (**b**). Operands are color coded as in Figure 3.

By connecting four MAC units, we obtained the MAC4 unit depicted in Figure 4, which updates the scalar *c* with the dot-product of vectors a and b of size four,

(3) c←c+a·b.

**Figure 5:**
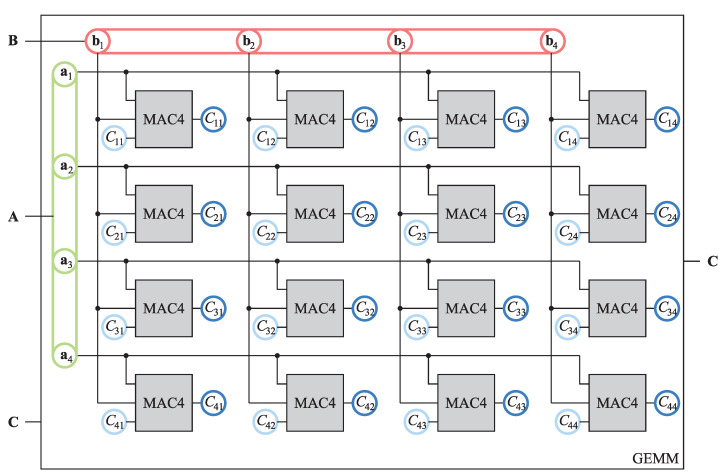
The GEMM unit composed of 16 MAC4 units. Matrix A is decomposed into four row vectors $a_{1},\ldots ,a_{4}$ and matrix B into four column vectors $b_{1},\ldots ,b_{4}$. Matrix C and its update are presented elementwise. Operands are color coded as in Figure 3.

By employing 16 MAC units, we implemented the GEMM unit presented in Figure 5, which performs the GEMM operation over four-by-four matrices.

The GEMM unit is a complex circuit that employs 64 multipliers and adders to perform the GEMM operation in one clock cycle. Note that we had to provide four-by-four matrices A and B, which equals thirty-two scalars, to compute the sixteen dot-products of matrix C. Thus, we needed to transfer only two scalars per one dot-product from the memory on each update, which is far more efficient compared eight scalars per dot-product in the case of one MAC4 unit.

For efficient implementation in hardware, we used a 16-bit signed fixed-point representation of the operands. To further improve the design’s area, speed, and energy efficiency, we approximated the arithmetic operations at the expense of introducing some error to the computation. The approximation of multipliers is much more beneficial, as they are much more expensive circuits than adders. Besides, the recent results for approximate computing suggest keeping adders exact, thus ensuring proper convergence of the accumulations [81]. Hence, the proposed approximate general matrix multiplication (AGEMM) unit utilizes approximate multipliers and exact adders.

### 3.2. Synthesis Results

We analyzed and compared the hardware performance of the 16-bit signed fixed-point GEMM and AGEMM units in terms of the power, area, delay, and power delay product (PDP). We compared the GEMM unit using the exact radix-4 multipliers to the AGEMM units employing the logarithmic multipliers DR-ALM5 [57] and TL16-8/4 [61], the nonlogarithmic multiplier RAD1024 [55], and the hybrid HRALM3 multiplier [62].

**Figure 6:**
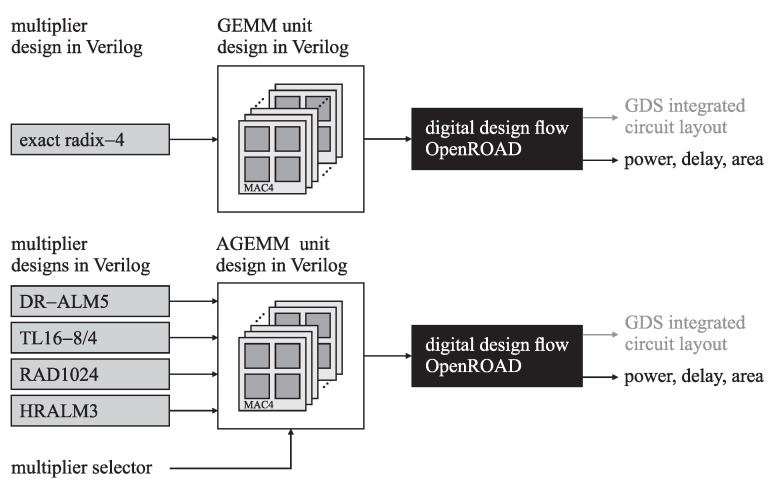
GEMM and AGEMM unit design workflow.

Following the schemes in Figure 4 and Figure 5, we implemented the units for the GEMM operation in the Verilog hardware description language. The design was modular and supported integrating any 16-bit signed fixed-point multiplier given in Verilog; let it be the exact radix-4 multiplier or a state-of-the-art approximate multiplier, as shown in Figure 6. Even though the unit’s core design was equal for all multipliers, we differentiated between the GEMM unit with the exact multiplier and the AGEMM unit with an approximate multiplier for clarity. To evaluate the design, we drove the unit’s Verilog code to the OpenROAD digital design flow [82], an open-source end-to-end Verilog, to the GDS compiler using the 45 nm Nangate Open Cell Library. We used timing with a 10 MHz virtual clock, a 5% signal toggle rate, and an output load capacitance of 10 fF to evaluate the power. The design flow resulted in a unit’s circuit layout and the following metrics: estimated power, delay, and area.

Table 1 shows the hardware metrics of the synthesized units. The study [62] showed that a single exact radix-4 multiplier has a delay of 1.74 ns, a power delay product (PDP) of 0.12 pJ, and an area of $1.58\times 10^{3}\;{\mu m}^{2}$. GEMM units are much more expensive circuits; the delay of the exact GEMM unit was 2.7-times longer, and it took 70-times more area and consumes 200-times more energy than the exact multiplier. The AGEMM units using the DR-ALM5 and the TL16-8/4 multipliers delivered lower energy consumption (PDP) than those using the RAD1024 and HRALM3 multipliers. Besides, the AGEMM unit with the DR-ALM5 multipliers possessed the shortest delay.

## 4. Honeybee Detection

Honeybees are crucial for terrestrial ecosystems due to their ability to pollinate plants and crops. Therefore, it is essential to continuously monitor their condition in beehives and provide the needed treatments. To lower the effort required for a beekeeper to determine the honeybees’ condition and to minimize the possible damage to the colony, it is vital to use an autonomous surveillance system able to detect and count honeybees in real time. This section evaluates the usability of the proposed AGEMM units in honeybee detection with the YOLOv4-tiny convolutional neural network. Firstly, we estimated the required configuration of the AGEMM units that would make real-time honeybee detection feasible. Secondly, we present the honeybee dataset, the experimental setup, and the proposed system’s detection results and conclude with the discussion.

### 4.1. YOLOv4-Tiny Inference with the GEMM Unit

The YOLOv4-tiny convolutional neural network, briefly described in Section 2.1, consists of 21 multiplication intensive convolutional layers and four layers without multiplications. We assumed that activation functions of the convolutional layers were given in terms of lookup tables and did not involve multiplications. To profit most from the GEMM and AGEMM units, we had to utilize them efficiently in the convolutional layers.

#### 4.1.1. A Convolutional Layer

A convolutional layer takes an input tensor and a set of multichannel filters to compute an output tensor. By convolving the input tensor consisting of *C* channels of *I*-by-*I* elements with a *C*-channel filter of *F*-by-*F* elements, we obtained one *O*-by-*O* channel of the output tensor. The number of channels in the output tensor equals the number of filters *K*. With the filter stride *S*, we adjusted the granularity of the convolution, which could result in a modified size of the output channels.

**Figure 7:**
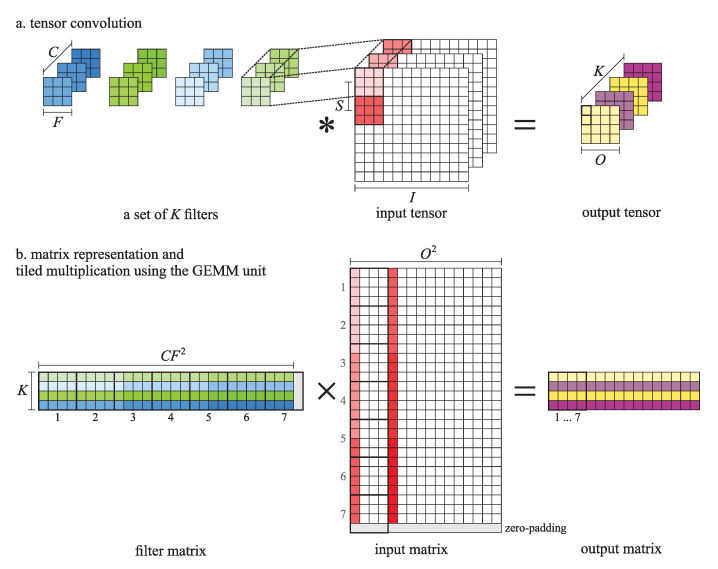
Tensor convolution (**a**) and its matrix equivalent with tiled matrix multiplication using the GEMM unit (**b**). The filter matrix and the input matrix are zero-padded to conform to the tile size.

As illustrated in Figure 7a, we computed a dot-product of a multichannel filter and a patch of the input tensor elements of size $CF^{2}$ to obtain one element of the output tensor. To obtain all elements of the output tensor, we computed $KO^{2}$ dot-products, performing $CF^{2}KO^{2}$ multiplications altogether.

#### 4.1.2. Computation with the AGEMM Unit

To use the GEMM unit in the convolution, we first laid out the filters and the input tensor to matrices and then performed the convolution through matrix multiplication. We put each filter to one line of the filter matrix and each patch of the input tensor to one column of the input matrix. The matrix multiplication of the *K*-by-$CF^{2}$ filter matrix and the $CF^{2}$-by-$O^{2}$ input matrix resulted in the *K*-by-$O^{2}$ output matrix, from which we could easily construct the output tensor. Further on, we partitioned the matrices into nonoverlapping four-by-four tiles; when necessary, we zero-padded the matrices to conform to the tile size. We computed each tile of the output matrix by successively applying the GEMM unit on the corresponding tiles of the filter matrix and the input matrix, as illustrated in Figure 7b. The output matrix consisted of $\left\lceil K/4\right\rceil \;\cdot \;\left\lceil O^{2}/4\right\rceil$ tiles. To compute one tile, we employed the GEMM unit $\left\lceil CF^{2}/4\right\rceil$ times.

Table 2 lists the parameters of the YOLOv4-tiny model layers and the number of GEMM operations needed to compute the output. For one inference pass through the YOLOv4-tiny backbone, we needed $58.85\times 10^{6}$ invocations of the GEMM unit. Considering delay through the GEMM unit in Table 1, we could theoretically make one inference pass of the YOLOv4-tiny backbone in 277 ms by using one unit only. With the concurrent use of eight units in a setup similar to the graphics processing units [66,83], we could reduce the processing times to 35 ms, leading to a theoretical 29 images per second, which should suffice for real-time object detection.

### 4.2. Honeybee Dataset

**Figure 8:**
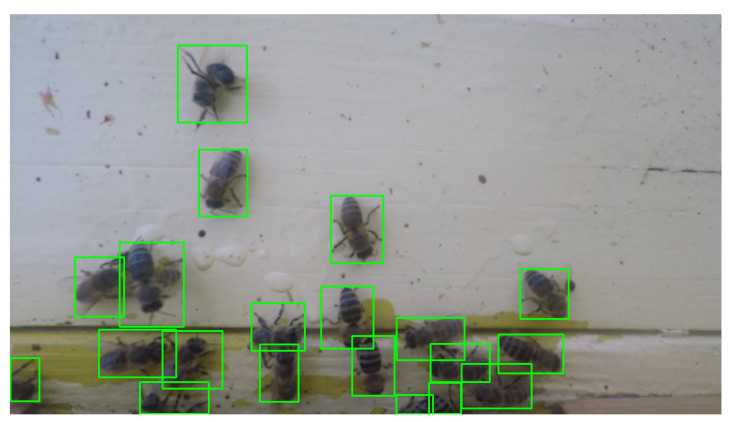
A sample image from the dataset with Carniolan grey honeybees and ground truth bounding boxes.

The images containing honeybees [84] were extracted from the video recorded at the Botanic Garden of the University of Ljubljana, where a beehive with a colony of Carniolan grey bees (*Apis mellifera carnica*), the native Slovenian breed, was placed. We set the camera above the beehive entrance and recorded the honeybees entering and exiting the hive using the shelf in front of the beehive entrance. With such a setup, we ensured a noninvasive recording of the honeybees in their natural environment. The dataset contained 65 images of size 2688-by-1504 pixels. There was a total of 1040 ground truth bounding boxes containing Carniolan grey honeybees. Figure 8 shows a sample image from the dataset with Carniolan grey honeybees and the ground truth bounding boxes.

### 4.3. Experimental Setup

**Figure 9:**
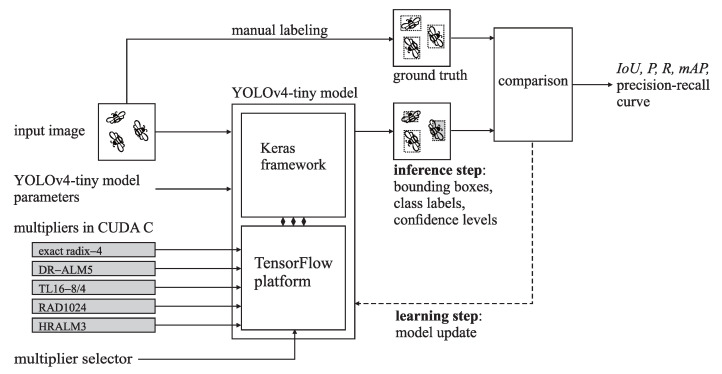
Object detection workflow with GEMM/AGEMM unit simulation.

Figure 9 illustrates the honeybee detection workflow. We used the YOLOv4-tiny model implementation from [85], which utilizes Keras [86], a deep learning framework for Python running on top of the TensorFlow [87] machine learning platform. We added the support for the approximate multipliers by replacing the floating-point multipliers in TensorFlow with the approximate fixed-point ones implemented in CUDA C. To attain basic image recognition features and alleviate data scarcity, we initialized the YOLOv4-tiny model with the weights [77], pretrained on the COCO dataset [88].

We randomly split the honeybee dataset into five sets of 13 images to perform the five-fold cross-validation using four sets for training and one for testing. We trained the neural network for 80 epochs with one image per batch [85] using the RMSprop optimizer with an initial learning rate of 0.01 and cosine annealing. We rescaled each original image to 416-by-416 pixels to suit the size of the input layer of the YOLOv4-tiny model (Table 2). To gear up the model for the approximate arithmetic and assure its convergence, we used the approximate fixed-point multiplication in the inference step and the floating-point multiplication in the learning step. We quantified the floating-point weights and inputs to the signed fixed-point representation with *f* fractional bits as $\left\lfloor v\cdot 2^{f}\right\rfloor /2^{f}$, where *v* is a floating-point value. We set f=12, which gave the lowest accuracy degradation for the exact radix-4 multiplier.

For each detection, the YOLOv4-tiny model predicted the bounding box and the class label, along with the confidence level. Only predictions with confidence levels greater than some predefined threshold, in our case 0.5, were returned. The quality of the object detection model depended on its ability to localize an object by determining its bounding box and classifying the contents to a predefined class label. The prediction was considered as a true positive if the predicted label was equal to the ground truth label and the intersection-over-union measure,

(4) $$IoU\left(B,G\right)=\frac{\mathrm{area}(B\cap G)}{\mathrm{area}(B\cup G)}\;,$$

where *B* is the predicted and *G* is the ground truth bounding box, equal to or greater than some threshold *t*.

The performance of an object detection model can be assessed in terms of precision and recall [89],

(5) $$\begin{array}{c} P=\frac{TP}{TP+FP}\;,\;R=\frac{TP}{TP+FN}\;, \end{array}$$

where TP, FP, and FN are the numbers of true positive, false positive, and false negative detections. Intuitively, precision *P* measures the accuracy of assigning the correct class label, while recall *R* measures the accuracy of finding ground truth objects.

By sorting the detections by the descending confidence level and incrementally calculating the precision and recall, we obtained a precision–recall curve. A good object detector should exhibit high precision and recall, with precision remaining high with increasing recall. One can roughly assess the performance of an object detector by computing the area under the precision–recall curve. To estimate the area under the curve, the average precision uses *N*-point interpolation [89],

(6) $$\begin{array}{c} AP=\frac{1}{N}\sum_{i=1}^{N}P_{\mathrm{interp}}\left(R_{i}\right)\;,\;P_{\mathrm{interp}}\left(R_{i}\right)=\max_{\tilde{R}\geq R_{i}}P\left(\tilde{R}\right)\;. \end{array}$$

The mean average precision metric, mAP, used in this paper, is the average of the 101-point interpolated AP metric over a set of thresholds *t*. The higher the value of the mAP∈[0,1] metric, the better the detector is. In the further analysis, we used the mAP[0.5] metric at the threshold t=0.5 and the mAP[0.5:0.95] metric averaged over ten equidistant thresholds t∈[0.5,0.95]. Using mAP-based metrics in combination with cross-validation is a standard approach for performance evaluation and model comparison in object detection benchmarks [2,89,90,91].

### 4.4. Object Detection Results

**Figure 10:**
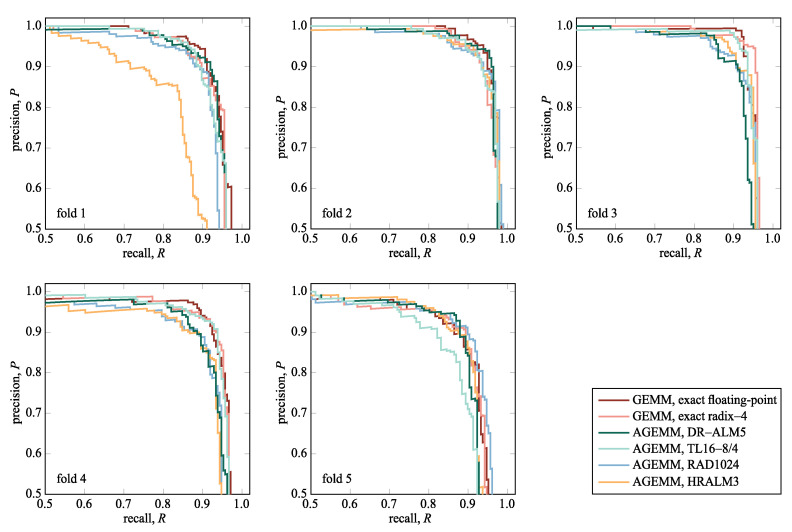
The precision–recall curve of the YOLOv4-tiny detector empowered with the selected GEMM and AGEMM units. Each plot presents curves obtained from the object detection for one of the cross-validation folds.

Figure 10 shows the precision–recall curves of the YOLOv4-tiny detector empowered with the GEMM and the AGEMM units for each of the five folds, and Table 3 reports the values of the mAP[0.5] and mAP[0.5:0.95] metrics averaged over five folds. A large area under the precision–recall curves indicates high precision and high recall. Precision drops on the far right side of the plot, where the confidence levels are low. Besides, high values of the mAP[0.5] metric indicate that the object detector performs well, while lower values of the mAP[0.5:0.95] metric suggest that the detector is not very good at localization.

Comparing the fixed-point and floating-point GEMM units revealed that the mAP[0.5] values of the detector with the fixed-point and floating-point units were equal. In contrast, the mAP[0.5:0.95] metric showed slightly worse localization of the fixed-point detectors.

Object detectors with AGEMM units performed similarly well as the object detectors with the fixed-point GEMM unit. Slightly worse results of the AGEMM unit using the HRALM3 multiplier could be attributed to the poorer performance in the first fold.

The estimated propagation delays of the GEMM and AGEMM units from Table 1 and the number of the GEMM unit invocations from Table 2 define the theoretical lower bound of the YOLOv4-tiny backbone execution time. Table 3 reports the backbone execution time of eight parallel GEMM or AGEMM units and the speedup, obtained as the ratio of the GEMM and AGEMM execution times.

### 4.5. Discussion

We evaluated four AGEMM units employing different state-of-the-art approximate multipliers. The comparison of the synthesis results in terms of the PDP for the approximate multipliers in Figure 2 and the AGEMM units in Table 1 revealed that the hardware characteristics of approximate multipliers dictated the hardware performance of the corresponding AGEMM units. The hardware characteristics favored the AGEMM units employing small approximate logarithmic multipliers.

The precision–recall curves in Figure 10 and the values in Table 3 showed that in honeybee detection, the majority of AGEMM units stood in line with the exact GEMM unit. The results revealed that the YOLOv4-tiny object detector did not favor one AGEMM unit over another, as all AGEMM units offered almost similar detection results, allowing replacing the exact GEMM unit. Thus, AGEMM units with considerable gains in hardware metrics are preferable. For real-time object detection, the execution time is an important metric, where AGEMM units with small approximate logarithmic multipliers excelled. The AGEMM unit using the DR-ALM5 multiplier could process up to 30% more images in the same time interval as the exact GEMM unit. Thus, only eight parallel AGEMM units using the DR-ALM5 multiplier sufficed to perform the YOLOv4-tiny backbone inference step up to 38-times per second, more than enough for real-time honeybee detection.

However, it is essential to note that the choice of the AGEMM unit is application-specific. In the presented honeybee detection problem, one should choose between the AGEMM unit using the TL16-8/4 multiplier, which is the best choice when optimizing the system for die-area, and the AGEMM unit employing the DR-ALM5 multiplier, which possesses the shortest propagation delay and lowest energy consumption.

The synthesis and detection results proved our hypothesis that the AGEMM units can efficiently replace the exact ones. For the good performance of object detectors, it is essential to use the AGEMM units in the inference step during the training, thus helping the deep neural network model adapt the weights in such a way to compensate for error introduced in the computation. Hence, the employment of more accurate, but, at the same time, more complex approximate multipliers in the AGEMM unit did not necessarily provide any significant improvement in object detection.

When an application can compensate inaccuracies in the computation, the AGEMM units could bring considerable gains of up to a 25% shorter execution time, 60% smaller chip area, 70% lower power usage, and 75% reduction in energy consumption. Furthermore, the results in object detection suggested that we could probably empower the AGEMM unit with even simpler approximate multipliers, obtained either by decreasing the bit-width or accuracy.

## 5. Conclusions

Currently, the trend in edge computing is to empower sensors near data sources with artificial intelligence features. Commonly, we execute the inference in the pretrained artificial intelligence models on the edge device’s graphics processing units. Recent graphics processing units contain special arithmetic units capable of performing intensive matrix–matrix multiply operations needed for the models. Convolutional neural network-based object detection applications need to work locally on an edge device for a fast response. The real-time inference in convolutional neural network models involves a vast number of matrix–matrix multiply operations, requiring additional processing power, which can compromise the edge device’s autonomy.

We proposed and designed the approximate general matrix multiply (AGEMM) unit for object detection. The AGEMM unit utilized approximate computing, a popular strategy for decreasing energy consumption and the overall complexity of arithmetic circuits. In particular, it performed a four-by-four matrix multiplication using approximate fixed-point multipliers and accurate fixed-point adders. We anticipate that applying the proposed AGEMM unit to convolutional neural network models could provide a significant benefit in terms of speed, area, and energy consumption at the expense of reduced accuracy, which we can compensate during the network’s training.

We implemented in Verilog four variants of AGEMM units using state-of-the-art approximate multipliers. To assess the hardware characteristics of the AGEMM units, we synthesized them with the 45 nm Nangate Open Cell Library. The best AGEMM unit using the DR-ALM5 multiplier reduced the propagation delay by 25%, the area by 60%, and the energy consumption by more than 75% compared to an accurate fixed-point GEMM unit.

We evaluated the usability of the proposed AGEMM unit by deploying it in the YOLOv4-tiny object detector specifically trained for honeybee detection. We used the detector implemented in the Keras/TensorFlow framework, where we replaced the exact floating-point multipliers with the approximate fixed-point multipliers. The accuracy of the detectors with the fixed-point multipliers was only slightly lower than the detector’s accuracy with the floating-point multiplier. Among the detectors employing the AGEMM units, we obtained the best results for the detector utilizing the approximate DR-ALM5 multiplier. The results also revealed that the use of more accurate and more expensive nonlogarithmic and hybrid multipliers cannot be justified in AGEMM units employed in convolutional neural network object detectors.

The high throughput of the proposed AGEMM unit suggested that YOLOv4-tiny could perform the inference in real time on a video with more than 30 frames per second using eight concurrent AGEMM units in a setup similar to that in recent graphics processing units. Moreover, the AGEMM unit’s exceptional energy efficiency promises that its employment in graphics processing units could lead to a prolonged autonomy of edge devices used in object detection.

We proved our hypothesis that the proposed AGEMM unit performed well in object detectors and brought significant savings in hardware and energy. The neural network could successfully compensate the approximation in the multiplications. Moreover, the obtained results indicated that we could probably use even shorter bit-width multipliers. Hence, we plan to employ eight-bit approximate multipliers and assess their usability in object detection, resulting in even faster processing and more savings in energy. We employed AGEMM units only in the inference step for detection and classification. As the efficiency of a network training process is as important as its inference, we plan to address the challenges of applying AGEMM units in training. We expect that AGEMM units would contribute to more efficient training and allow the pretrained deep neural network to adapt its weights at runtime.

The following abbreviations are used in this manuscript:

## Figures and Tables

**Table 1:** The synthesis results of 16-bit unsigned fixed-point GEMM and AGEMM units.

Unit	Multiplier	Delay (ns)	Power (mW)	Area $\left(\cdot 10^{3}\;\mu m^{2}\right)$	PDP (pJ)
GEMM	exact radix-4	4.70	5.32	107.3	25.0
AGEMM	DR-ALM5 [57]	3.58	1.58	43.2	5.6
	TL16-8/4 [61]	4.16	1.48	39.0	6.2
	RAD1024 [55]	3.78	2.83	61.9	10.7
	HRALM3 [62]	4.46	1.80	45.7	8.0

**Table 2:** Configuration of the YOLOv4-tiny model backbone layers with the number of GEMM operations per layer. Additional configuration details are available in the file /cfg/yolov4-tiny.cfg in the repository [77].

Layer	Type	Input		Filters			Output	Operations
		Size	Number	Size	Stride	Number	Size	
		I	C	F	S	K	O	
1	c	416	3	3	2	32	208	605,696
2	c	208	32	3	2	64	104	3,115,008
3	c	104	64	3	1	64	104	6,230,016
4	c	104	64	3	1	32	104	3,115,008
5	c	104	32	3	1	32	104	1,557,504
6	c	104	64	1	1	64	104	692,224
7	p	104	128	2	2	128	52	-
8	c	52	128	3	1	128	52	6,230,016
9	c	52	128	3	1	64	52	3,115,008
10	c	52	64	3	1	64	52	1,557,504
11	c	52	128	1	1	128	52	692,224
12	p	52	256	2	2	256	26	-
13	c	26	256	3	1	256	26	6,230,016
14	c	26	256	3	1	128	26	3,115,008
15	c	26	128	3	1	128	26	1,557,504
16	c	26	256	1	1	256	26	692,224
17	p	26	512	2	2	512	13	-
18	c	13	512	3	1	512	13	6,340,608
19	c	13	512	1	1	256	13	352,256
20	c	13	256	3	1	512	13	3,170,304
21	c	13	512	1	1	255	13	352,256
22	c	13	256	1	1	128	13	88,064
23	u	13	128	2	0.5	128	26	-
24	c	26	384	3	1	256	26	9,345,024
25	c	26	256	1	1	255	26	692,224
Legend: c, convolutional; p, pooling; u, upsampling.								58,845.696

**Table 3:** Honeybee detection using the GEMM unit with the exact floating-point and fixed-point multipliers and the AGEMM unit with the selected approximate multipliers. The reported values of the mAP measures are the means and standard deviations averaged over five folds. The estimated execution time is given for eight parallel GEMM or AGEMM units.

Unit	Multiplier	*mAP*[0.5]	*mAP*[0.5:0.95]	Execution	Speedup
				Time [ms]	
GEMM	exact float	0.94 ± 0.02	0.46 ± 0.01	–	–
	exact radix-4	0.94 ± 0.02	0.43 ± 0.02	34.6	1.00
AGEMM	DR-ALM5 [57]	0.93 ± 0.02	0.43 ± 0.01	26.3	1.31
	TL16-8/4 [61]	0.93 ± 0.03	0.42 ± 0.03	30.6	1.13
	RAD1024 [55]	0.93 ± 0.02	0.42 ± 0.03	27.8	1.24
	HRALM3 [62]	0.91 ± 0.04	0.40 ± 0.04	32.8	1.05

## Data Availability

The data presented in this study are openly available in “Carnolian Grey Honeybees Dataset”, IEEE Dataport, doi: https://doi.org/10.21227/b6cx-ak33 (accessed on 20 May 2021).
